# Hypoparathyroidism in an Egyptian child with Hutchinson-Gilford progeria syndrome: a case report

**DOI:** 10.1186/1752-1947-6-17

**Published:** 2012-01-17

**Authors:** Kotb Abbass Metwalley Kalil, Hekma Saad Fargalley

**Affiliations:** 1 Department of Pediatrics, Faculty of Medicine, Assiut University, Assiut, Egypt

## Abstract

**Introduction:**

Hutchinson-Gilford progeria syndrome is a rare genetic disorder. It is reported to be present in one in eight million and is characterized by severe growth failure, early loss of hair, lipodystrophy, scleroderma, decreased joint mobility, osteolysis, early atherosclerosis and facial features that resemble those of an aged person. Apart from diabetes mellitus, there are no reported abnormalities of thyroid, parathyroid, pituitary or adrenal function. Here, we report the case of a 10-year-old Egyptian child with Hutchinson-Gilford progeria syndrome and hypoparathyroidism.

**Case presentation:**

A 10-year-old Egyptian boy was referred to our institution for an evaluation of recurrent attacks of muscle cramps, paresthesia of his fingertips and perioral numbness of two months duration. On examination, we found dilated veins present over his scalp with alopecia and frontal bossing, a beaked nose, thin lips, protruding ears, a high pitched voice with sparse hair over his eyebrows and eyelashes and micrognathia but normal dentition. His eyes appeared prominent and our patient appeared to have poor sexual development. A provisional diagnosis of progeria was made, which was confirmed by molecular genetics study. Chvostek's and Trousseau's signs were positive. He had low total calcium (5.4 mg/dL), low ionized calcium (2.3 mg/dL), raised serum phosphate (7.2 mg/dL), raised alkaline phosphatase (118 U/L) and low intact parathyroid hormone (1.2 pg/mL) levels. He was started on oral calcium salt and vitamin D; his symptoms improved with the treatment and his serum calcium, urinary calcium and alkaline phosphates level were monitored every three months to ensure adequacy of therapy and to avoid hypercalcemia.

**Conclusion:**

Routine checking of serum calcium, phosphorus and parathyroid hormone will help in the early detection of hypoparathyrodism among children with progeria.

## Introduction

Progeria is a rare combination of dwarfism and premature aging characterized by thin, atrophic shiny skin with sclerodermoid changes, sparse to absent hair of the scalp, eyebrows and eyelashes, craniofacial disproportion, a sculpted beaked nose, short stature, a pyriform thorax, thin limbs and musculoskeletal and cardiovascular manifestations [1]. The classic form is known as Hutchinson-Gilford syndrome. It occurs sporadically, with a reported incidence of one in eight million births and male predominance with male to female ratio of 1.5:1 [2]. Hypoparathyroidism is an inherited or acquired deficiency of the parathyroid hormone (PTH) or its action [3]. Hypoparathyroidism occurs in all age groups. The classic clinical presentation (Chvostek's or Trousseau's sign, tetany, seizures, cardiac failure) is attributed to hypocalcemia [4].

## Case presentation

A 10-year-old Egyptian boy was referred to our institution for an evaluation of recurrent attacks of muscle cramps, paresthesia of the fingertips and perioral numbness of two months duration. Apart from drug treatment for hypercholesterolemia, he was not receiving any other medication. He was the second-born male child to consanguineous Egyptian parents, with the other sibling being normal. His prenatal history was uneventful. He was delivered at term by Caesarean delivery and cried immediately after birth. He was apparently normal until two years of age. His mental development was normal. No other family members were affected with similar complaints. His mother noticed that he was not gaining weight and had decreased scalp hair growth. On examination, his weight was 11.5 kg (below the third centile), his height was 102 cm (below the third centile) and his head circumference was 52 cm. He had prominent dilated veins present over his scalp with alopecia and frontal bossing, a beaked nose, thin lips, protruding ears, a high pitched voice, sparse hair over the eyebrows and eyelashes, micrognathia but normal dentition and his eyes appeared prominent. There were multiple patches of coarse and thickened skin, especially over the dorsum of his hands and shoulders. He appeared to have poor sexual development. The terminal ends of his fingers appeared broad and stubby and his nails were dystrophic. His limbs were thin with bilateral contracture of both knee joints. Based on his history and the clinical findings, a provisional diagnosis of progeria was made. The diagnosis was confirmed genetically by detection of mutations (G608G) in the laminin A gene (*Lmna*). He had no signs of rickets. His blood pressure was normal. Chvostek's and Trousseau's sign were positive. He had no hypopigmentation of his skin or oral thrush. Investigations revealed that a hemogram, liver function tests, renal function tests, his lipid profile, fasting blood sugar, sodium, potassium and magnesium levels, blood gases, a urinary aminoacidogram, thyroid function tests, an electrocardiogram, an echocardiogram, an abdominal ultrasound, an electroencephalogram and a computerized tomography scan of his brain were all normal. He had low total calcium 5.4 mg/dL (normal range, 8.5 mg/dL to 10.5 mg/dL), low ionized calcium 2.3 mg/dL (normal range, 4.5 mg/dL to 5.6 mg/dL), raised serum phosphate 7.2 mg/dL (normal range, 2.4 mgdL to 4.1 mg/dL), raised alkaline phosphatase 118 U/L (normal range, 30 U/L to 95 U/L) and low intact PTH 1.2 pg/mL (normal range, 12 pg/mL to 72 pg/mL). A radiograph of his hands and feet revealed resorption of the terminal phalanges with osteoporosis. He was started on oral calcium salt and vitamin D; his symptoms improved with the treatment and his serum calcium, urinary calcium and alkaline phosphates levels were monitored every three months to ensure adequacy of therapy and to avoid hypercalcemia.

## Discussion

We are reporting this case to draw attention to the rare clinical association of hypoparathyroidism with progeria. To the best of our knowledge, no case with a similar association has been published online. The term progeria is derived from the Greek word *geras*, meaning old age [5]. In 1886, Hutchinson described the first patient and in 1887, Gilford described a second patient with similar clinical findings [6]. In this syndrome, the rate of aging is accelerated up to seven times that of normal. This is due to various abnormalities of mesodermal tissue and decreased survival time of fibroblasts [7]. *De novo *mutations of *Lmna*, which encodes for a major constituent of the inner membrane lamina, have been reported [8].

Though the majority of reported cases have shown an autosomal dominant inheritance pattern, factors such as paternal and maternal ages have also shown to influence the inheritance of this disease [9]. About 97% of children affected are Caucasian, but all children with Hutchinson-Gilford progeria have a similar appearance, regardless of their racial or ethnic background. The average life span is 13 years (range, seven years to 27 years) with occasional survival till the age of 45 years [10]. Death is mainly due to cardiovascular complications like myocardial infarction or congestive heart failure [2]. There is no effective treatment to date. The only available treatment option uses a multidisciplinary approach towards symptomatic treatment and timely identification and prompt management of complications. Affected children are normal at birth and grow normally until about the end of the first year, when both normal growth and weight gain slow down. At the end of the first decade, the size attained is that of a normal child of three years of age. Loss of hairs and subcutaneous fat along with sclerodermatous changes give rise to a characteristic 'plucked bird' appearance at about six to 12 months of age. The affected child has a short stature and is underweight, with an average height of 100 cm and average weight of 12 kg to 15 kg or less [11]. The patient in our case had the classical features, which included facial features, failure to thrive, alopecia, poor sexual maturation and normal intelligence. The normal level of cholesterol in our case may be attributed to the treatment with cholesterol-lowering medication since the age of three years. The association of our patient's symptoms and physical findings of hypocalcemia and hyperphosphatemia with a low PTH are consistent with the diagnosis of hypoparathyroidism. Merideth *et al. *reported an elevated serum phosphorus level in 8 out of 15 children with progeria. On the other hand, they did not report any data about the levels of serum calcium or PTH [12]. Although attempts to correct hypocalcaemia and hyperphosphatemia with calcium and vitamin D are beneficial, the long-term benefits in our patient are not known. Treatment of hypoparathyroid patients with synthetic PTH is controversial and its effectiveness in our patient is questionable as well. The later onset of hypoparathyrodism associated with nail dystrophy raises the possibility of an autoimmune process affecting our patient. The reported association of diabetes mellitus and progeria supports our idea regarding autoimmune process that can affect children with progeria.

## Conclusion

Routine checking of serum calcium, phosphorus and PTH will help in the early detection of hypoparathyrodism among children with progeria.

## Consent

Written informed consent was obtained from the patient's parents for publication of this case report and any accompanying images. A copy of the written consent is available for review by the Editor-in-Chief of this journal.

## Competing interests

The authors declare that they have no competing interests.

## Authors' contributions

KA and HS carried out the patient diagnosis, investigation, follow-up, management and the final manuscript writing. Both authors read and approved the final manuscript.
